# HOPE PARTIALLY MEDIATES STRESS AND PERCEIVED BURDEN IN FAMILY CAREGIVERS OF PERSONS LIVING WITH A DEMENTIA

**DOI:** 10.1093/geroni/igac059.2747

**Published:** 2022-12-20

**Authors:** Jocelyn McGee, Dennis Myers, Rebecca Meraz, Clay Polson, Weiming Ke, Angela McClellan

**Affiliations:** Baylor University, Waco, Texas, United States; Baylor University, Waco, Texas, United States; Baylor University, Waco, Texas, United States; Baylor University, Waco, Texas, United States; Baylor University, Waco, Texas, United States; Baylor University, Waco, Texas, United States

## Abstract

Recent research suggests that hope may positively impact perceived burden in family caregivers of persons living with a dementia. However, there are few studies that have examined hope, as a multidimensional construct, on perceived burden. The purpose of this study was to investigate the relationship between objective and subjective stressors, hope and perceived burden in a sample of caregivers using taking into consideration multiple hope dimensions—specifically hope-agency and hope-pathway. Hierarchical multiple regression and mediation analyses were utilized in a sample of one-hundred and fifty-five family caregivers. Multiple regression analysis revealed that low hope-agency predicted high levels of perceived burden after controlling for known burden correlates. Hope-pathway did not impact perceive burden. Objective stress on perceived burden was partially mediated by hope-agency. The multi-dimensional aspects of hope should be taken into consideration when assessing this population. Hope-related psychosocial interventions aimed at bolstering multiple aspects of hope among family caregivers of persons with a dementia should be further developed and assessed in clinical intervention research.

